# Curcumin Loaded in Niosomal Nanoparticles Improved the Anti-tumor Effects of Free Curcumin on Glioblastoma Stem-like Cells: an In Vitro Study

**DOI:** 10.1007/s12035-020-01922-5

**Published:** 2020-05-19

**Authors:** Sajad Sahab-Negah, Fatemeh Ariakia, Mohammad Jalili-Nik, Amir R. Afshari, Sahar Salehi, Fariborz Samini, Ghadir Rajabzadeh, Ali Gorji

**Affiliations:** 1 Neuroscience Research Center, Mashhad University of Medical Sciences, Mashhad, Iran; 2 Department of Neuroscience, Faculty of Medicine, Mashhad University of Medical Sciences, Mashhad, Iran; 3 Shefa Neuroscience Research Center, Khatam Alanbia Hospital, Tehran, Iran; 4 Student Research Committee, Mashhad University of Medical Sciences, Mashhad, Iran; 5 Department of Medical Biochemistry, Faculty of Medicine, Mashhad University of Medical Sciences, Mashhad, Iran; 6 Department of Pharmacology, Faculty of Medicine, Mashhad University of Medical Sciences, Mashhad, Iran; 7 Natural Products and Medicinal Plants Research Center, North Khorasan University of Medical Sciences, Bojnurd, Iran; 8 Department of Food Nanotechnology, Research Institute of Food Science and Technology, Mashhad, Iran; 9 Department of Materials and Metallurgical Engineering, Materials and Metallurgical Engineering Faculty, Semnan University, Semnan, Iran; 10 Department of Neurosurgery, Faculty of Medicine, Mashhad University of Medical Sciences, Mashhad, Iran; 11 Department of Neurosurgery and Department of Neurology, Westfälische Wilhelms-Universität, 48149 Münster, Germany; 12 Epilepsy Research Center, Westfälische Wilhelms-Universität Münster, 48149 Münster, Germany

**Keywords:** Brain tumor, Cytokine, Cellular engineering, Cell death, Glioma

## Abstract

Using a novel curcumin-loaded niosome nanoparticle (CM-NP), the present study was designed to evaluate the effect of curcumin on human glioblastoma stem-like cells (GSCs). CM-NP has a diameter of ~ 60 nm and a zeta potential of ~ − 35 mV with a constant physicochemical stability. The cytotoxic effects of free curcumin (CM) and CM-NP were investigated on GSCs obtained during the removal of a brain tumor. Both CM and CM-NP caused a dose-dependent decrease in cell proliferation and viability of GSCs. The IC50 values of CM and CM-NP on GSCs were 50 and 137 μg/ml after 24 h, respectively. CM-NP exerted significantly higher effects on GSC viability, apoptosis, cell cycle arrest, and the expression of Bax, a pro-apoptotic marker, compared with CM. In addition, the migration of GSCs was significantly impaired following the administration of CM-NP compared with CM. Furthermore, CM-NP significantly increased the values of reactive oxygen species and decreased the mRNA expressions of NF-κB and IL-6 of GSCs compared with CM. Our data also revealed that CM-NP could significantly reduce the invasiveness of GSCs compared with CM, possibly via MCP-1-mediated pathways. In addition, CM-NP exhibited a significantly greater inhibitory effect on colony formation of GSCs compared with CM. These data indicate that CM-NP exhibited stronger anti-tumor effects on GSCs than CM. Although further in vivo investigations are warranted, our results suggest that CM-NP could be an ideal carrier to deliver curcumin for potential therapeutic approaches into glioblastoma.

## Introduction

Glioblastoma multiform (GBM) is the most prevalent and lethal malignant primary brain tumor originating from glial cells, representing one-third of the whole central nervous system tumors [1]. Current therapeutic options available for GBM have limited efficacy, offering short-term survival improvement with a considerable side effects. Thus, improvement in available therapeutic approaches and the implementation of novel treatments are warranted. GBM contains functional subsets of tumorigenic and self-renewing stem cells called glioblastoma stem-like cells (GSCs). The characteristic features of GSCs, such as infiltrative property, resistance to various treatments, and progressive nature, strongly suggested their key role in tumor initiation, invasion, and recurrence [2]. GSCs are identified as a novel target for the treatment of resistant tumor cells and prevention of cancer recurrence [3, 4].

Curcumin, a natural compound in turmeric, has been traditionally believed to exert desirable therapeutic effects on several chronic neurological diseases [5, 6]. Both experimental studies and clinical trials point to anti-neoplastic effects of curcumin [7, 8]. Numerous investigations suggest that curcumin may be used as an adjuvant substance to augment available treatments of GBM [9, 10]. The chemotherapeutic effects of curcumin on different GBM cell lines indicate implication of a variety of signaling pathways and molecular targets, including (i) induction of autophagy via suppression of the Akt/mammalian target of rapamycin and activation of extracellular signal-regulated kinase pathways [11–13], (ii) inhibition of cell growth, migration, and invasiveness through the modulation of the matrix metalloproteinases (MMP), and inhibition of the Janus kinase (JAK)/signal transducers and activators of transcription (STAT) 3 signaling pathway [14–17], and (iii) modulation of tumor invasion, angiogenesis, and metastasis via upregulation of apoptotic pathways and induction of G2/M phase arrest as well as inhibition of nuclear factor κB (NF-κB) and phosphoinositide 3 kinase [18, 19] Furthermore, curcumin inhibits patient-derived GSC viability and proliferation as well as sphere- and colony-forming potentials, possibly via a reactive oxygen species (ROS)–dependent pathway as well as the JAK/STAT3 pathway [20]. However, the therapeutic potential of curcumin is limited due to its rapid metabolism as well as indigent water solubility and absorption [21].

Nanotechnology-based therapeutic delivery systems, including nanoparticles, nano-emulsions, and liposomes, have emerged to promote the bioavailability, low aqueous solubility, cellular uptake, and anti-tumor activity of curcumin [5, 22], which possibly improve targeted delivery and cellular internalization of the curcumin nanoparticle into the tumor cells [6]. Various biodegradable polymers of natural or synthetic origin have been used for curcumin nano-encapsulation [23–25]. The effects of different nano-sized particles of curcumin have been examined in GBM cells in both in vitro studies and clinical trials [26, 27]. Curcumin nanoparticles have been shown to suppress the growth of multiple GBM cell lines through the reduction of stem-like tumor cells [28, 29]. In an attempt to improve the targeting property for efficient curcumin therapeutic approaches on GBM, we designed a novel curcumin-loaded niosome system to evaluate its effect on GSCs. Niosomes, composed of a combination of a non-ionic surfactant and lipid compounds with an overall neutral charge [30, 31], are biodegradable, biocompatible, non-immunogenic, and safe for delivery of both hydrophilic and hydrophobic drugs [31]. Niosomes have the ability to overcome the blood-brain barrier (BBB) and raise the stability and concentrations of the encapsulated medication within the brain [32].

The majority of prior studies have focused on the effect of curcumin on GBM cell lines and limited investigations evaluated its effect on GSCs obtained during tumor surgery. In this in vitro study, we assessed whether curcumin-encapsulated noisome nanoparticle (CM-NP) can exert considerably greater water solubility and systemic bioavailability than free curcumin. Furthermore, the effect of CM-NP on proliferation, survival, migration, and invasive properties of GSCs was compared with free curcumin. Finally, the signal transduction pathways mediating CM-NP and free curcumin effects on GSCs were investigated.

## Materials and Methods

### Study Design

**Fig. 1:**
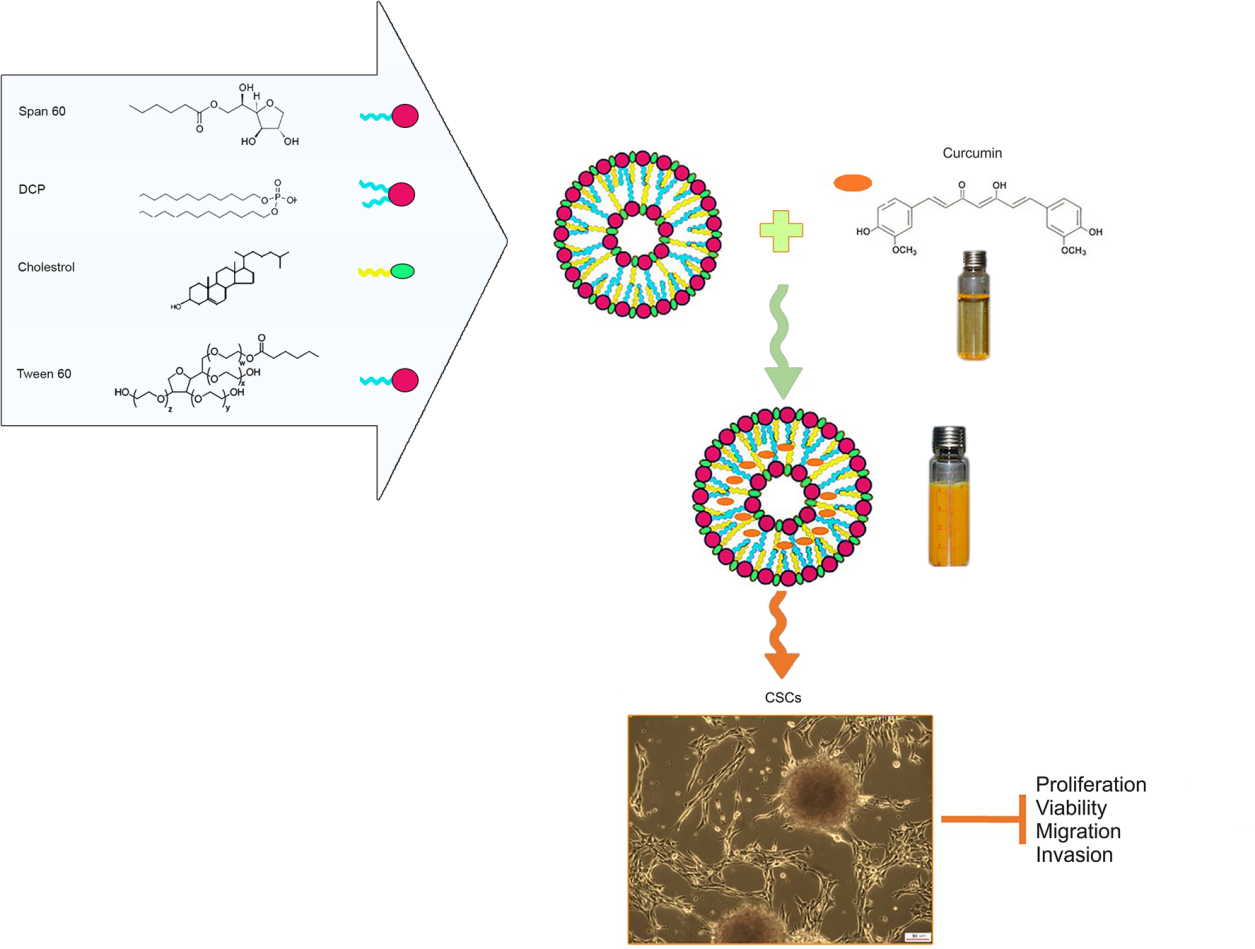
A schematic overview of synthetic procedure of the nano-niosome-loaded curcumin, its delivery path, and its ability of targeting glioblastoma stem-like cells (GSCs). The niosomal carrier (Tween 60-Span 60-cholestrol) enhanced the entrapment efficiency of curcumin and di-acetyl phosphate (DCP) was used for loading curcumin as well as increasing stability and efficiency. Curcumin was encapsulated in the shell of niosome. Anti-tumor activity of the nano-niosome-loaded curcumin was proved by the efficient reduction of the viability, proliferation, migration, and invasion of human GSCs

Niosome nanoparticles were prepared using the thin-film hydration method. Formation of niosomes requires an amphiphilic molecule composed of two main parts, a hydrophilic head group and a hydrophobic tail. Curcumin was encapsulated in the shell of niosome nanoparticles and the characteristics of CM-NP were assessed. To determine the anti-tumor activity of CM-NP, GSCs were cultured and cultivated with CM-NP. The effects of CM-NP on different properties of GSCs were evaluated (Fig. 1).

### Preparation of CM-NP

Niosome formulation was synthesized using the thin-film hydration method with some modifications, as described previously [33, 34]. Briefly, sorbitan monostearate (Span 60), Tween 60 (1:2 M ratio), cholesterol, and dicetyl phosphate (all obtained from Sigma, Germany) were weighted and dissolved in 10 ml of ethanol-chloroform mixture (1:2 *v*/*v*). The organic solvent was evaporated in a rotary evaporator (Rotavapor® R-114, BUCHI, USA) in order to form a thin film in the round bottom flask at 60 °C. The thin film was rehydrated with phosphate-buffered saline (PBS) at pH 7.4. Subsequently, to obtain a uniform niosome, the hydrated niosome was sonicated using a sonicator (sonopuls HD-3200, Bandelin, Germany) with 90% amplitude, 150 W power, and 20 kHz frequency for 5 min (30 s on and 10 s off). To encapsulate curcumin, 100 μM of curcumin (Sigma, USA) was dissolved in chloroform-ethanol mixture along with niosome. After preparation, samples were stored at 4 °C for further experiments.

### Morphology Analyses

#### Size and Zeta Potential Assessments

The mean size and size distribution of niosomes were assessed using Zeta-sizer Nano ZS (Malvern Instruments Ltd., UK) with a helium-neon laser at 630 nm in room temperature. Briefly, niosomal samples were appropriately diluted (1:10) with deionized water and the size of the sample was measured based on dynamic light scattering (DLS) method. The zeta potential of niosomes was detected by the abovementioned instrument. The size distribution was measured by the polydispersity index. Niosomal samples were filtered through 0.45-μm membrane filters (Satrious, Germany) to avoid multiple scattering. The real refractive and the imaginary refractive indices were set at 1.33 and 0.01, respectively.

#### Transmission Electron Microscopy (TEM)

To analyze the effective formation of the vesicles of nanoparticles, TEM was used. The sample was diluted (1:10) with deionized water to decrease the vesicle concentration. A drop of 2% uranyl acetate was immediately added to the diluted sample for 2 min and sonicated for 1 min in an ultrasound bath at room temperature. A drop of this solution was located on a copper mesh for 2 min. After another min, the excess solution was removed by a filter paper and the grid surface was air-dried at room temperature. Finally, TEM images of nanoparticles were taken using a Leo 912 OMEGA transmission electron microscope (Germany).

### Determination of Entrapment Efficiency

Entrapment efficacy (EE) was performed by spectrophotometry. First, different concentrations of curcumin in methanol were applied to set calibration curve. Then, 5 ml niosome suspension was loaded on an Amicon MPS (Millipore, USA) filtration tube and centrifuged at 6000 rpm (Hettich Tuttlingen, Germany). Entrapped niosome remained at the top of the tube membrane and supernatant was separated in the bottom filter cup. The concentration of free curcumin in the supernatant was measured by absorbance measurement at 426 nm. The EE% was calculated by

$$ \mathrm{EE}\%=\frac{\mathrm{Total}\ \mathrm{amount}\ \mathrm{of}\ \mathrm{curcumin}-\mathrm{free}\ \mathrm{curcumin}\ }{\mathrm{Total}\ \mathrm{amount}\ \mathrm{of}\ \mathrm{curcumin}}\times 100 $$

### In Vitro Curcumin Release

In vitro drug release profile of curcumin-loaded niosome nanoparticles was performed by the direct dispersion method as described before [35]. A known amount of CM-NP (150 μg/ml) was dispersed in 33 ml PBS at pH 7.4 and was divided equally in 33 microfuge tubes. A thermostable water bath was used to keep the tubes at 37 °C. Free curcumin was insoluble in water; thus, the solution was shaken (100 rpm, 10 min) to separate the released curcumin from the loaded nanoparticles at predetermined time intervals (0, 2, 4, 6, 24, 48, 72, 84, 96, 120, 132, 156, 180, 204 h). The released curcumin was re-dissolved in methanol and the absorbance was measured using spectrophotometer at 426 nm and the values of the released curcumin were measured by a standard curve of curcumin in methanol.

### Fourier Transform Infrared Spectroscopy (FTIR)

To assess the compatibility of curcumin with niosome, a FTIR machine (Thermo Nicolet) was used to determine the unloaded and loaded curcumin in niosome. The samples were then scanned between the range of 4000 and 400 cm^−1^ with a resolution of 4 cm^−1^.

### Differential Scanning Calorimetry (DSC)

Calorimetric analyses were performed using a differential scanning calorimetry (Eirsa, Iran). The equipment was calibrated by indium. In all experiments, the reference pan was empty. Firstly, 14 mg of niosome (loaded and unloaded) was transferred from the bulk to the aluminum crucible and calorimetric analysis was done using a heating scan from 5 to 350 °C at a scan rate of 5 °C/min at nitrogen atmosphere. After the calorimetric characterization of each sample, the interaction between components was assessed.

### GSC Culture

The tissue sample was collected during tumor resection from a 55-year-old man who suffered from GBM. Informed consent was obtained from the patient. The experimental protocol was evaluated and approved by the Ethical Committee of Shefa Neuroscience Research Center, Tehran, Iran. The tissue was mechanically dissociated and enzymatically digested to a single-cell suspension. The GSCs were grown in Dulbecco’s modified Eagle’s medium/F12 medium (Gibco, Germany), 2% fetal bovine serum (Sigma, Germany), 100 units/ml of penicillin-streptomycin (Sigma, Germany), 2% B27 (Gibco, Germany), 1% L-glutamine (Sigma, Germany), 1% N2 (Gibco, Germany), 20 ng/ml epidermal growth factor (Sigma, Germany), and 20 ng/ml basic fibroblast growth factor (Sigma, Germany) at 37 °C in a humidified incubator (5% CO_2_). The medium was changed twice a week. Cells between passages 4 and 8 were used for the experiments.

### Cell Proliferation Assay

The cell proliferation was evaluated by MTT assay (Atocel, Austria). GSCs (1 × 10^4^/well) were seeded in 96-well culture plates and kept overnight. These cells were incubated with curcumin and CM-NP for the next 24 or 48 h. After that, 10 μl of the MTT solution in PBS (5 mg/ml) was added to each well at a final concentration of 0.05%. After 3–4 h, the supernatant was removed and to dissolve the formazan crystals, 100 μl of dimethyl sulfoxide (Sigma, Germany) was added to each well. Then, the microplates were gently shaken in the dark for 60 min and the absorbance was assessed between 545 and 630 nm by a Stat FAX303 plate reader.

### Viability Assay

The viability of GSCs was evaluated using LIVE/DEAD Viability/Cytotoxicity kit (Invitrogen, Molecular Probes, USA). GSCs (2 × 10^4^ per well) were seeded in a 96-well culture plate for 24 h. After overnight incubation, the medium was replaced with medium containing curcumin (50 μg/ml) and CM-NP (137 μg/ml). After 24 h incubation, the culture medium was discarded and 100 μl of the stain solution (containing 2 μM Calcein AM and 4 μM of EthD-1 in PBS) was added for 30 min at 37 °C. Images of live/dead cells were taken using a fluorescent microscope (Axiovert 200, Zeiss, Germany). The mean percentage of live cells was measured after analysis carried out by ImageJ software.

### Cytotoxicity Assay

The cytotoxicity of curcumin and CM-NP were determined by the CytoTox-Fluor™ Cytotoxicity kit (Promega, Germany). The test measured dead-cell activity with crossing the fluorogenic peptide substrate (bis-alanyl-alanyl-phenylanlanyl-rhodamine 110) into the dead cells and giving the fluorescence signal [36]. GSCs (1 × 10^4^ per well) were cultured in medium with half maximal inhibitory concentration (IC50) of curcumin and CM-NP for 24 h at 37 °C. After overnight incubation, cytotoxicity assay reagent in an equal volume was added (100 μl per well) to all wells, mixed briefly by orbital shaking, and then incubated for at least 30 min at 37 °C. Cytotoxicity was measured using a fluorescent plate reader (485 nm Ex/535 nm Em; PerkinElmer VICTOR X5, USA). The relative number of dead cells was indicated by analysis the activity of dead-cell protease activity released from dead cells with no membrane integrity.

### Cell Cycle Analysis

GSCs were cultured in 6-well plates at a density of 1 × 10^6^ cells per well overnight. Curcumin (50 and 200 μg/ml) and CM-NP (137 and 411 μg/ml) were then added and allowed to incubate for 24 h. Cells were trypsinized, centrifuged at 2000 rpm for 5 min at 4 °C, suspended with ice-cold PBS, and fixed in 70% ethanol at − 20 °C overnight. After fixation, GSCs were washed and re-suspended with ice-cold PBS. Then, cells were incubated with RNase A (100 μl) for 30 min at room temperature. Next, the cells were re-suspended in 400 μl PI/Triton X-100 solution (50 μg/ml PI, 1 mg/ml sodium citrate, 0.1% Triton X-100) for 30 min in the dark. Next, cell cycle distribution was assessed from 10,000 cells in a BD FACSCALIBUR™ FLOW CYTOMETER (Becton Dickinson, USA). DNA cell cycle analysis of flow cytometry data was performed by the software FlowJo V10 (Flowjo, OH, USA).

### Determination of Cell Death by Annexin V/PI Staining

The quantity of apoptosis and necrosis induced by CM-NP against GSCs was measured by the Annexin V/PI staining kit (Cayman, USA). The GSCs were seeded at 1 × 10^6^ cells per well on a 6-well plate and treated with curcumin (50 and 200 μg/ml) and CM-NP (137 and 411 μg/ml) for 24 h. Next, cells were washed with 200 μl of 1× binding buffer and centrifuged at 400*g* for 5 min. After that, 50 μl of Annexin V-FITC and PI reagent were added to the cells. The cells were incubated for 10 min in the dark at room temperature. Next, the final volume was set at 200 μl with 1× binding buffer and the final volume was set at 250 μl with 1× binding buffer. The number of viable, early apoptotic, late apoptotic, and necrotic cells was quantified immediately by the BD FACSCALIBUR™ FLOW CYTOMETER (Becton Dickinson, USA). The results were analyzed using the software FlowJo V10 (Flowjo, USA).

### Evaluation of Intracellular ROS

Visualizing and quantitating the generation of ROS was performed by the DCFDA/H2DCFDA-cellular ROS detection assay kit according to the manufacturer’s protocols (Abcam, UK). The GSCs were seeded at 25 × 10^3^ cells per well on a 96-well dark-sided culture plate. Then, the cells were washed with 1× buffer and received 100 μl of H2DCFDA (25 μM) media solutions (45 min in the dark). Then, cells were washed with PBS and incubated with IC50 concentration of curcumin and CM-NP for 8 h. The mean fluorescence intensity generated by the H2DCFDA oxidation was evaluated at an excitation wavelength of 485 nm and an emission wavelength of 535 nm using a Victor X5 Multiplable Plate Reader (Perkin Elmer, USA). In addition, CM-NP-induced ROS activity was tested by fluorescent microscopy. Dissociated GSCs were then inserted on poly-L-lysine coated 96-well clear plates. IC50 concentrations of curcumin and CM-NP were added to cells for 4 h. The cells were washed with PBS and H2DCFDA fluorescence was added to the cell and incubated for 30 min. Images were obtained using a fluorescent microscope (Axiovert 200, Zeiss, Germany).

### Assessment of Gelatinases Using Gelatin Zymography

The secretions of two members of the matrix metalloproteinases, MMP-2 and MMP-9, in culture conditioned medium were analyzed using gelatin zymography [37]. Briefly, the GSCs were treated with different doses of curcumin (50 and 200 μg/ml) and CM-NP (137 and 411 μg/ml) for 24 h. Cultured media were centrifuged, the pellet was discarded, and 50 μg of total protein from the supernatant was electrophoresed on 12% separating sodium dodecyl sulfate polyacrylamide gel electrophoresis containing 0.1% (1 mg/ml) of gelatin. The gel was washed three times with washing buffer containing 2.5% Triton X-100 every 20 min for three times and then incubated for 24 h at 37 °C in the incubation buffer (2.5% Triton X-100 in 50 mM Tris pH 7.4, 5 mM CaCl2, 1 μM ZnCl2). The gel was stained with 0.5% Coomassie Brilliant Blue R-250, 40% ethanol, and 2% acetic acid in dH2O for 30 min, and then de-stained with 25% ethanol and 10% acetic acid in dH2O. The gelatinolytic activity (zones of gelatin degradation) was assessed by GS-800 calibrated densitometer (Bio-RAD, USA). The analysis was performed by using Image J 1.52a software (NIH, USA).

### Quantitative Real-time Polymerase Chain Reaction Assessments

Total RNA extraction of the treated cells (7 × 10^5^ cells per well, in 6-well plates) was performed according to the RNeasy® mini kit protocol (Qiagen, Germany). The quantification and quality control of extracting RNA was conducted in triplicate with a NanoDrop spectrophotometer (Thermo Fisher Scientific, Germany). Then, RNAs were reverse-transcribed using the RevertAid First Strand cDNA Synthesis (Thermo Fisher Scientific, Germany). The quantitative RT-PCR analysis was performed by RealQ Plus 2X MasterMix Green-without Rox™ (Amplicon, Denmark). Next, quantitative RT-PCR was carried out with specific primers for p53, Bax, Bcl-2, NF-κB, IL-6 (Santa Cruz, Germany), monocyte chemoattractant protein-1 (MCP-1), and CXCL-3 (Macrogene, Korea). The cDNA amplification was conducted using the Light-Cycler 96 real-time PCR system (Roche Applied Science, USA). Gene expression data were normalized to GAPDH. The 2^−ΔΔCt^ method was used to analyze the relative expression of target genes. The primer sequences (forward and reverse) are listed in Table 1.

**Table 1:** Summary of primers

Gene symbol	Gene name	Primers (5′ → 3′)
BAX	BCL2 associated X, apoptosis regulator	Forward: GGAGCTGCAGAGGATGATTG Reverse: CCAGTTGAAGTTGCCGTCAC
Bcl-2	BCL2 apoptosis regulator	Forward: CTGAGGAGCTTTGTTTCAACCA Reverse: TCAAGAAACAAGGTCAAAGGGA
p53	Tumor protein p53	Forward: ACCCTTGCTTGCAATAGGTG Reverse: AACAAAACACCAGTGCAGGC
MCP-1	Monocyte chemoattractant protein 1	Forward: CATGAAAGTCTCTGCCGCC Reverse: GGTGACTGGGGCATTGATTG
NF-κB	Nuclear factor-kappa B	Forward: GCGAGAGGAGCACAGATACC Reverse: CTGATAGCCTGCTCCAGG
CXCL3	Chemokine (C-X-C motif) ligand 3	Forward: CGCCCAAACCGAAGTCATAG Reverse: GCTCCCCTTGTTCAGTATCTTTT
*IL-6*	Interleukin 6	Forward: CCTGAACCTTCCAAAGATGGC Reverse: TTCACAAGGCAAGTCTCCTCA
GAPDH	Glyceraldehyde-3-phosphate dehydrogenase	Forward: ACAACTTTGGTATCGTGGAAGG Reverse: GCCATCACGCCACAGTTTC

### Migration Assay

GSCs migration was evaluated by a wound healing assay as described previously [38]. After 72-h incubation, GSCs reached 90% confluence. A straight line scratch on cell monolayer was done with a sterile 100-μl pipette tip. PBS washing was performed to remove the debris. The cells were then treated with curcumin (6.25 and 12.5 μg/ml) and CM-NP (17.12 and 34.25 μg/ml) and incubated for 72 h. The lesion border areas were observed and photographed using an inverted microscope (ZEISS Axiovert 200, Zeiss, Germany). GSCs migration distance was calculated at 4, 24, 48, and 72 h.

### Clonogenicity Assay

To evaluate anchorage-independent tumor growth potential, colony formation assay was conduct using soft agar (Merck, Germany). Single cells (15 × 10^3^ per well) were seeded on agarose-coated 96-well flat bottom plates. Colonies were formed in 3 days and then were treated with 50 μg/ml of curcumin as well as 137 μg/ml of CM-NP for 5 days. The images were taken by an inverted microscope (Axiovert 200, Zeiss, Germany).

### Immunohistochemistry

To study the effect of curcumin and CM-NP on the expression of brain tumor stem cell markers, immunocytochemistry was performed according to a previously described method [39]. Briefly, GSCs were grown on poly-D-lysine 96-well plates and treated with curcumin (25 μg/ml) and CM-NP (68.5 μg/ml) and incubated for 72 h. GSCs were washed with PBS and fixed with 4% formaldehyde for 30 min and then were permeabilized by incubation with 0.2% Triton X-100 (in PBS) for 10 min at room temperature. Non-specific bindings were blocked through incubation with 1.5% goat serum and 1% bovine serum albumin (Sigma, Germany) in PBS for 30 min at room temperature. Primary antibodies, including nestin (1:250, Abcam, USA) and Sox2 (1:250, Abcam, USA) as the markers for cancer stem cells, were used. Secondary antibody (goat anti-rabbit IgG conjugated to FITC) was applied for 1 h at 4 °C. Propidium iodide (Sigma-Aldrich, USA) was used to detect cell nuclei. Data were analyzed using fluorescent microscopy (ZEISS Axiovert 200, Zeiss, Germany).

### Statistical Analysis

Analyses were conducted by GraphPad Prism software (version 17). Statistically significant differences between different groups were assessed using one-way analysis of variance (ANOVA). Post hoc multiple comparisons were conducted by Tukey’s tests. Statistical analysis of migration assay data was performed by two-way repeated-measure ANOVAs with Tukey’s post hoc tests. Data were presented as the mean ± standard deviation and the significance level was considered at *P* < 0.05.

## Results

### Nano-drug Characterization

**Fig. 2:**
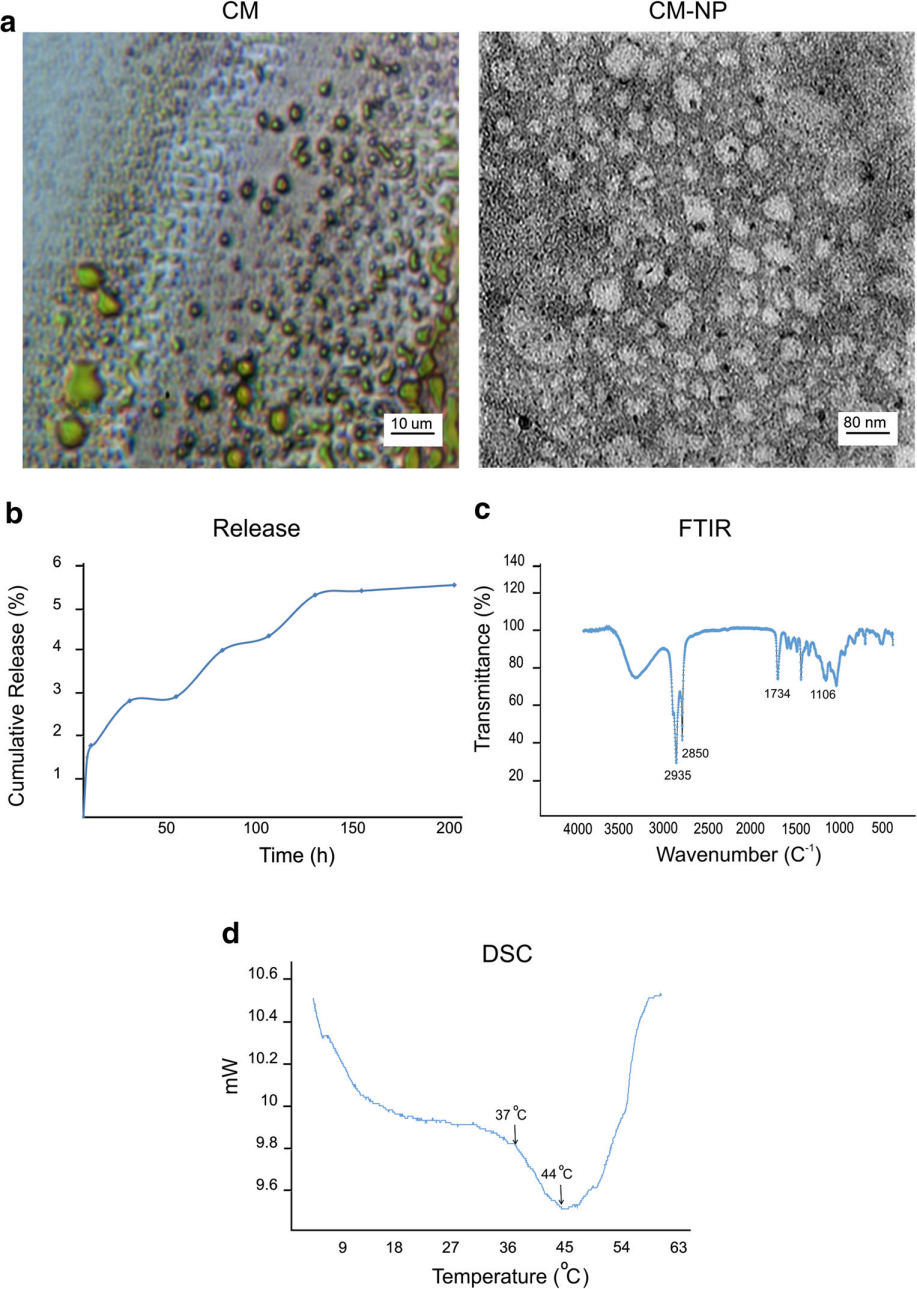
Characterization of curcumin-encapsulated noisome nanoparticle (CM-NP). **a** Size of curcumin (CM, left) and CM-NP (right) was measured by transmission electron microscopy (TEM). TEM images showed particles with a spherical morphology and an average size of 60 nm. **b** In vitro release profile of CM-NP was assessed in PBS at physiological pH (7.4). The release of CM from the niosome was ~ 14% at 24 h. **c** Interaction between components of CM-NP was evaluated by fourier transform infrared (FTIR) spectra. FTIR analysis indicated four peaks at 3860 cm^−1^, 2916 cm^−1^, 1738 cm^−1^, and 1104 cm^−1^, which were corresponding to cholesterol, Tween 60, Span 60, and C–O–C interactions, respectively. **d** Differential scanning calorimetry (DSC) thermograms of CM-NP were assessed between 5 and 60 °C. DSC analysis pointed to the stability of CM-NP at body temperature

To develop an effective curcumin delivery system, we constructed curcumin-loaded niosome nanoparticle using the thin-film hydration method. TEM images indicated that nanoparticles have a monodisperse size distribution with a diameter of ~ 60 nm (Fig. 2a). Using DLS method, the average size of CM-NP was ∼ 90 nm and the heterogeneity index was 0.2 ± 0.002. Successful formulation of CM-NP is further examined by the surface zeta potential of nanoparticles in deionized water. The zeta potential of niosome nanoparticles was ~ − 35 mV, indicating that the CM-NP has a good stability. The entrapment efficiency of CM-NP was ~ 80%. The in vitro release profile of the loaded curcumin from the niosome nanoparticles indicates an initial release of only 14.5% of curcumin after 24 h (Fig. 2b). Release profile of curcumin from niosome nanoparticles reached in a sustained pattern over time, indicating high stability of CM-NP.

To determine the interaction between different components of CM-NP, FTIR spectra were performed. As shown in Fig. 2c, FTIR spectrum showed the peaks at 3860 cm^−1^, 2916 cm^−1^, 1738 cm^−1^, and 1104 cm^−1^, which were corresponding to cholesterol, Tween 60, Span 60, and niosome formation of C–O–C interactions, respectively. Thermal characterization was also performed using differential scanning calorimetry. The DSC thermogram displayed a sharp endothermic peak at 45 °C, which corresponds to the melting of the CM-NP (Fig. 2d). DSC thermogram of CM-NP powder exhibited sufficient stability at 37 °C. These results revealed a significant interaction of curcumin with the shell structure of niosome, enhanced entrapment of curcumin into niosomal formulations, and sustained drug release.

### GSC Culture

**Fig. 3:**
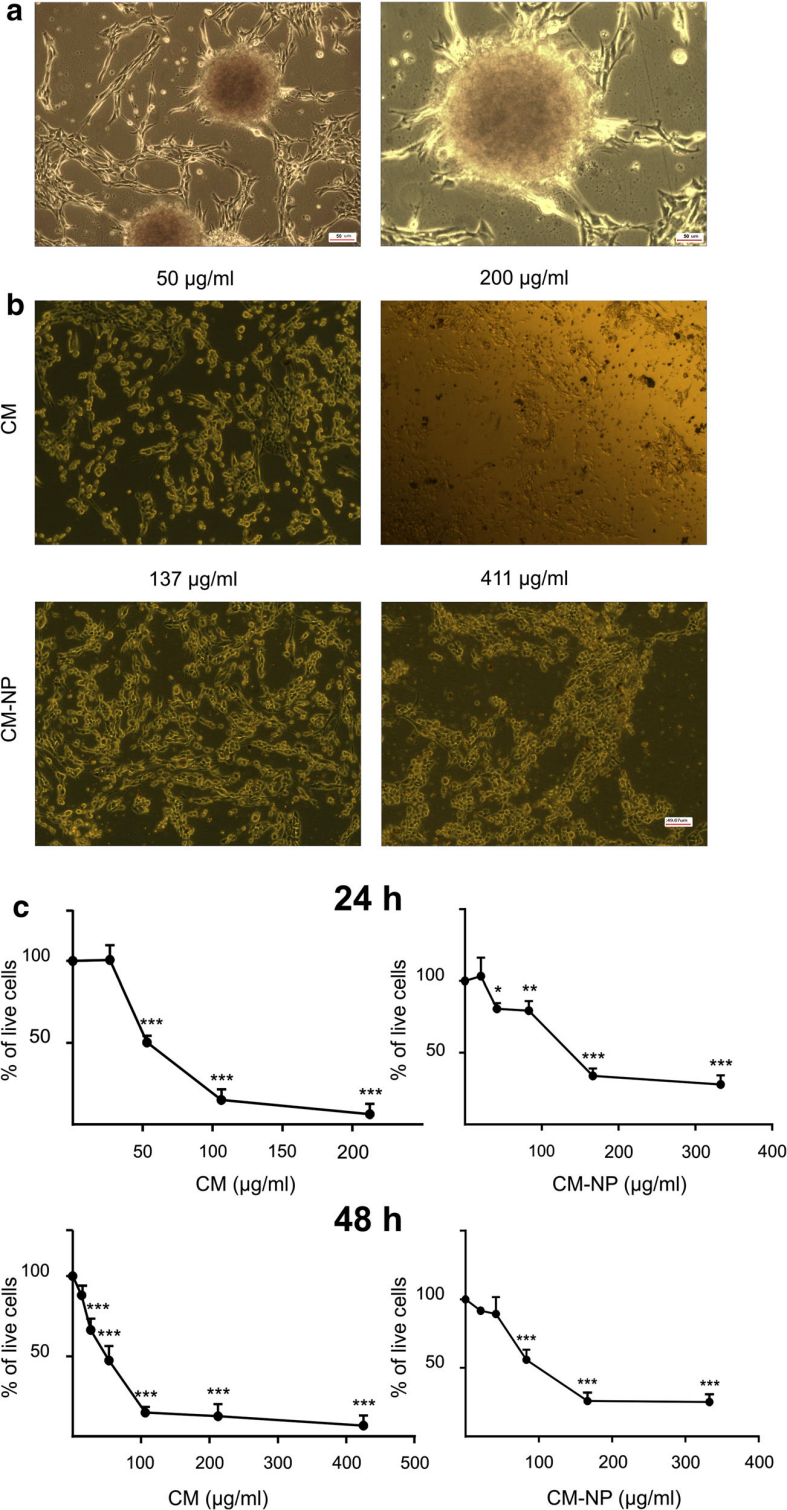
Cytotoxic effects of curcumin (CM) and curcumin-encapsulated noisome nanoparticle (CM-NP) on glioblastoma stem-like cells (GSCs). **a** Representative phase contrast micrographs of neurosphere formation of GSCs. **b** Phase contrast images of GSCs following 24-h treatment with CM (50 and 200 μg/ml) and CM-NP (137 and 411 μg/ml). **c** Cytotoxicity of CM and CM-NP on GSC proliferation was assessed by the MTT assay. To determine the half maximal inhibitory concentration (IC50) of drugs, the percentage of live GSCs was measured after 24- and 48-h treatment with different concentrations of CM and CM-NP. The data are presented as means ± SD. Single, double, and triple asterisks indicate *P* < 0.05, *P* < 0.01, and *P* < 0.001, respectively

To evaluate the self-renewal ability and the proliferation capacity, GSCs were passaged for 4 times (each time reached ~ 80% confluence). The tumor stem-like single cells produced spheres after 6 days, which could be passaged for 4–5 times, indicating self-renewal property of GSCs in spheres (Fig. 3a).

### Tumor Growth Results

#### CM-NP Inhibits Cell Proliferation and Viability of GSCs

The effects of different doses of curcumin and CM-NP on GSCs morphology were investigated. Application of curcumin and CM-NP treatment caused shrinkage and partial detachment of GSCs after 24 h, pointing to the cytotoxic effects of CM-NP (Fig. 3b). The functional effects of curcumin and CM-NP on cell proliferation of the GSCs were assessed by MTT assay. GSCs were incubated with different concentrations of curcumin and CM-NP and cell proliferation was assessed after 24 and 48 h. As shown in Fig. 3c, both curcumin and CM-NP significantly inhibited the proliferation of GSCs in a time- and dose-dependent manner. The IC50 value of CM-NP on GSCs was 137 μg/ml after 24 h, which decreased to 101 μg/ml after 48 h (Fig. 3c). Our results also showed that IC50 values of curcumin on GSCs were 50 μg/ml and 43 μg/ml after 24 and 48 h, respectively (Fig. 3c). For further experiments, mostly the values of 50 μg/ml and 137 μg/ml were chosen to investigate the effects of curcumin and CM-NP, respectively.

**Fig. 4:**
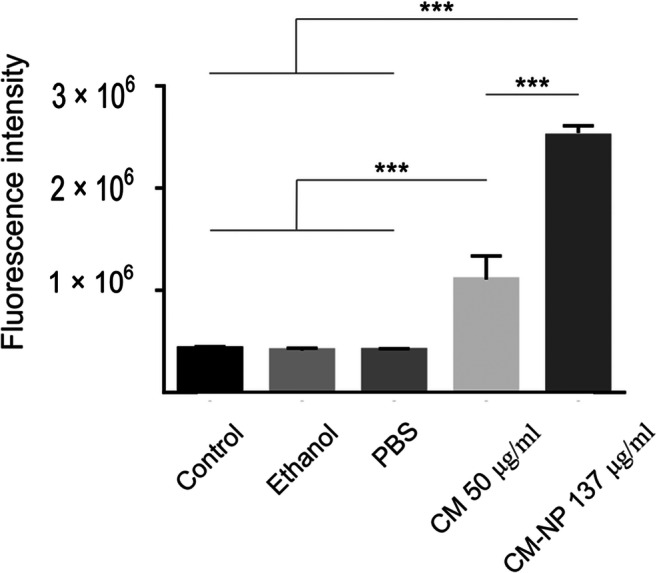
Analysis of cytotoxicity of curcumin (CM) and curcumin-encapsulated noisome nanoparticle (CM-NP) on glioblastoma stem-like cells (GSCs). The relative number of dead cells was identified by the amount dead-cell protease activity using a fluorogenic peptide substrate crossing into the dead cells. The values are expressed as mean ± SD. Triple asterisks indicate *P* < 0.001

**Fig. 5:**
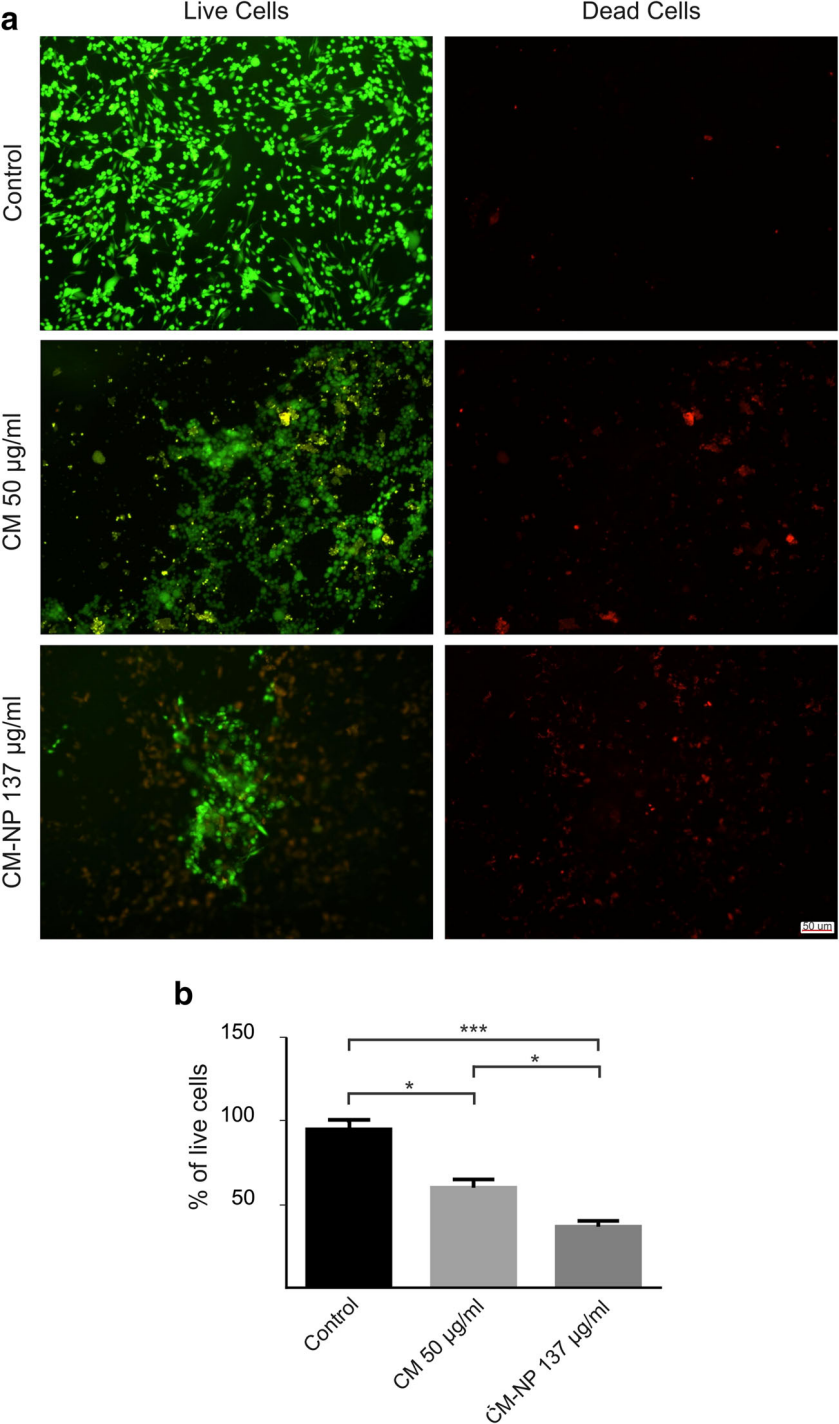
Live/dead assay of glioblastoma stem-like cells (GSCs) treated with IC50 concentration of curcumin (CM) and curcumin-encapsulated noisome nanoparticle (CM-NP). **a** Living cells are labeled green (Calcein AM) and dead cells are labeled red (ethidium homodimer) after treatment with CM (50 μg/ml) and CM-NP (137 μg/ml). **b** Quantitative analyses of GSC viability in different groups are shown. The values are expressed as mean ±. Single and triple asterisks indicate *P* < 0.05 and *P* < 0.001, respectively

**Fig. 6:**
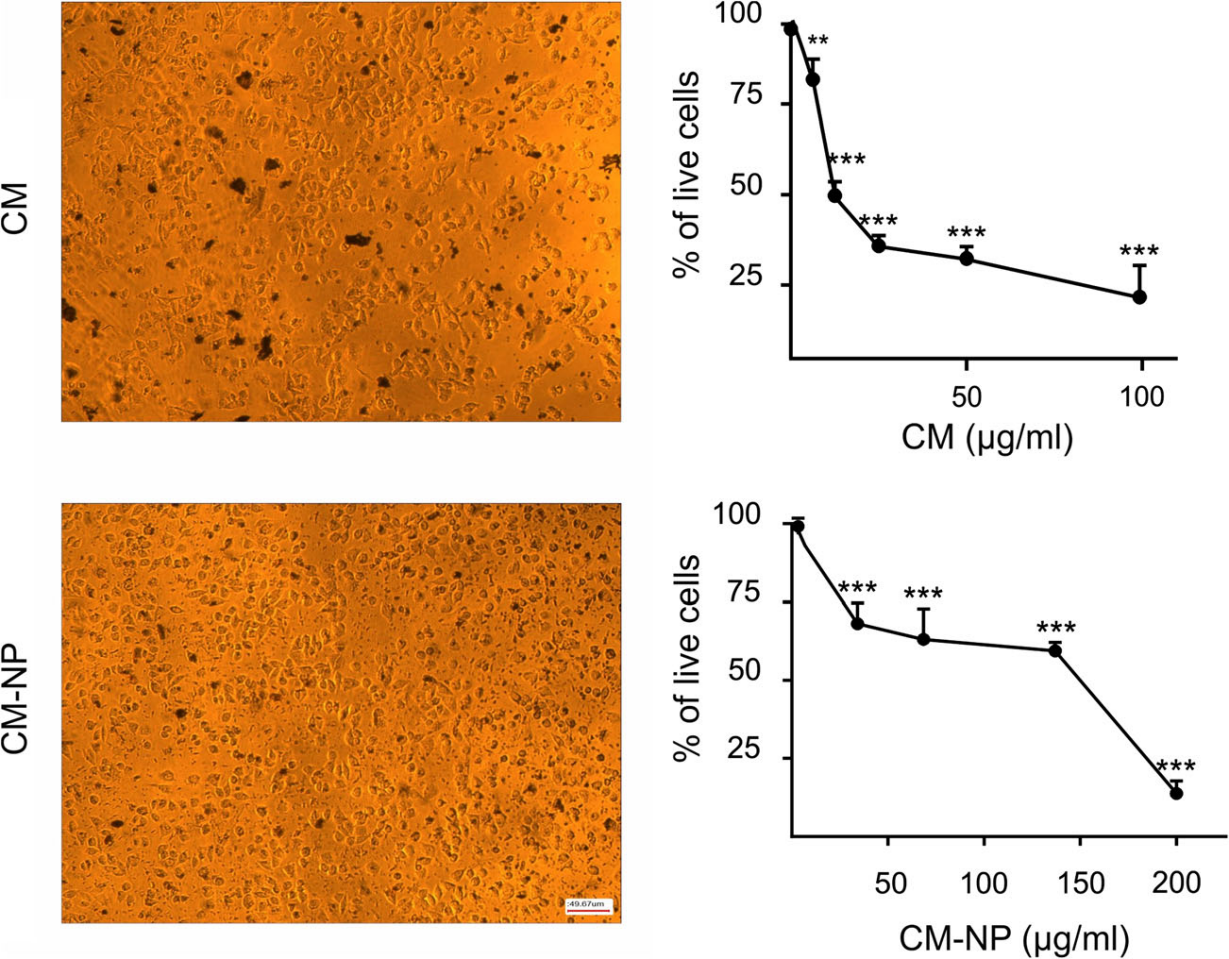
The effects of curcumin (CM) and curcumin-encapsulated noisome nanoparticle (CM-NP) on viability of NIH-3T3 cells. NIH-3T3 cells were incubated with different concentrations of CM and CM-NP for 24 h. The MTT assay was used to determine cell viability of CM- and CM-NP-treated cells. The data are presented as mean ± SD. Double and triple asterisks indicate *P* < 0.01 and *P* < 0.001, respectively

To detect the relative number of dead cells, the effects of curcumin and CM-NP on proteolytic activities associated with GSCs death were measured. CM-NP significantly enhanced the activity of proteases compared with free curcumin and the control groups (Fig. 4; *P* < 0.001). Furthermore, the cytotoxicity induced by curcumin and CM-NP on cell viability of GSCs was tested using live/dead assay (Fig. 5a). GSCs treated with curcumin and CM-NP were harvested after 24 h and were subjected to viability assay. Cell viability of GSCs significantly decreased after administration of CM-NP compared with curcumin (*P* < 0.05) and the control groups (Fig. 5b; *P* < 0.001). Furthermore, curcumin significantly decreased cell viability compared with the control group (Fig. 5b; *P* < 0.05). To establish whether curcumin and CM-NP at different doses were toxic to normal cells, NIH-3T3 cells were treated with these compounds for 24 h. Both curcumin and CM-NP exhibited dose-dependent effects on cell survival of NIH-3T3 cells. Administration of curcumin at a concentration above 12.5 μg/ml was associated with 50–80% loss of NIH-3T3 cells (Fig. 6). However, cell viability of NIH-3T3 cells following application of CM-NP at concentrations between 100 and 150 μg/ml was ~ 70% (Fig. 6).

#### CM-NP Induces Apoptosis and Necrosis in GSCs

**Fig. 7:**
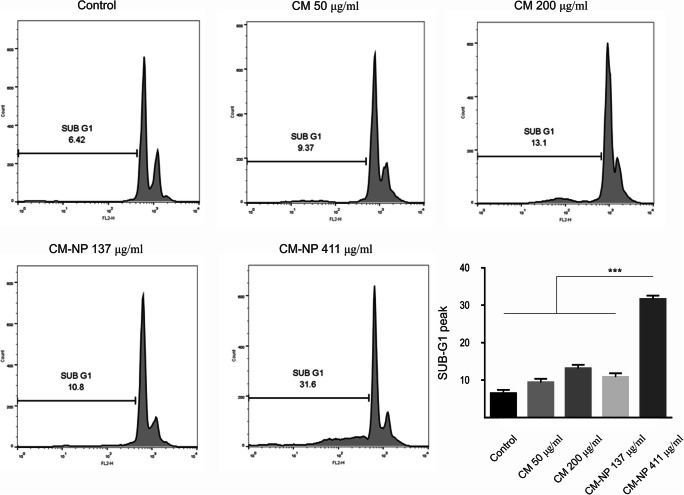
The effects of curcumin (CM) and curcumin-encapsulated noisome nanoparticle (CM-NP) on glioblastoma stem-like cells (GSCs) cell cycle. GSCs treated with different concentrations of CM and CM-NP for 24 h were stained with propidium iodide (PI) and the cell cycle was assessed using flow cytometry. The cell cycle was determined in GSCs treated with CM (50 and 200 μg/ml) and CM-NP (137 and 411 μg/ml) as well as in the control group (non-treated GSCs). Note the strong effect of CM-NP at 411 μg/ml on cell cycle arrest compared with the other groups. The data represent as the mean ± SD. Triple asterisks indicate *P* < 0.001

We further examined both cell cycle and apoptosis parameters in curcumin- and CM-NP-treated GSCs. Cell cycle analysis showed that the application of high doses of CM-NP on GSCs resulted in a significant higher accumulation of cells in sub-G1 phase compared with the other groups (Fig. 7; *P* < 0.001).

**Fig. 8:**
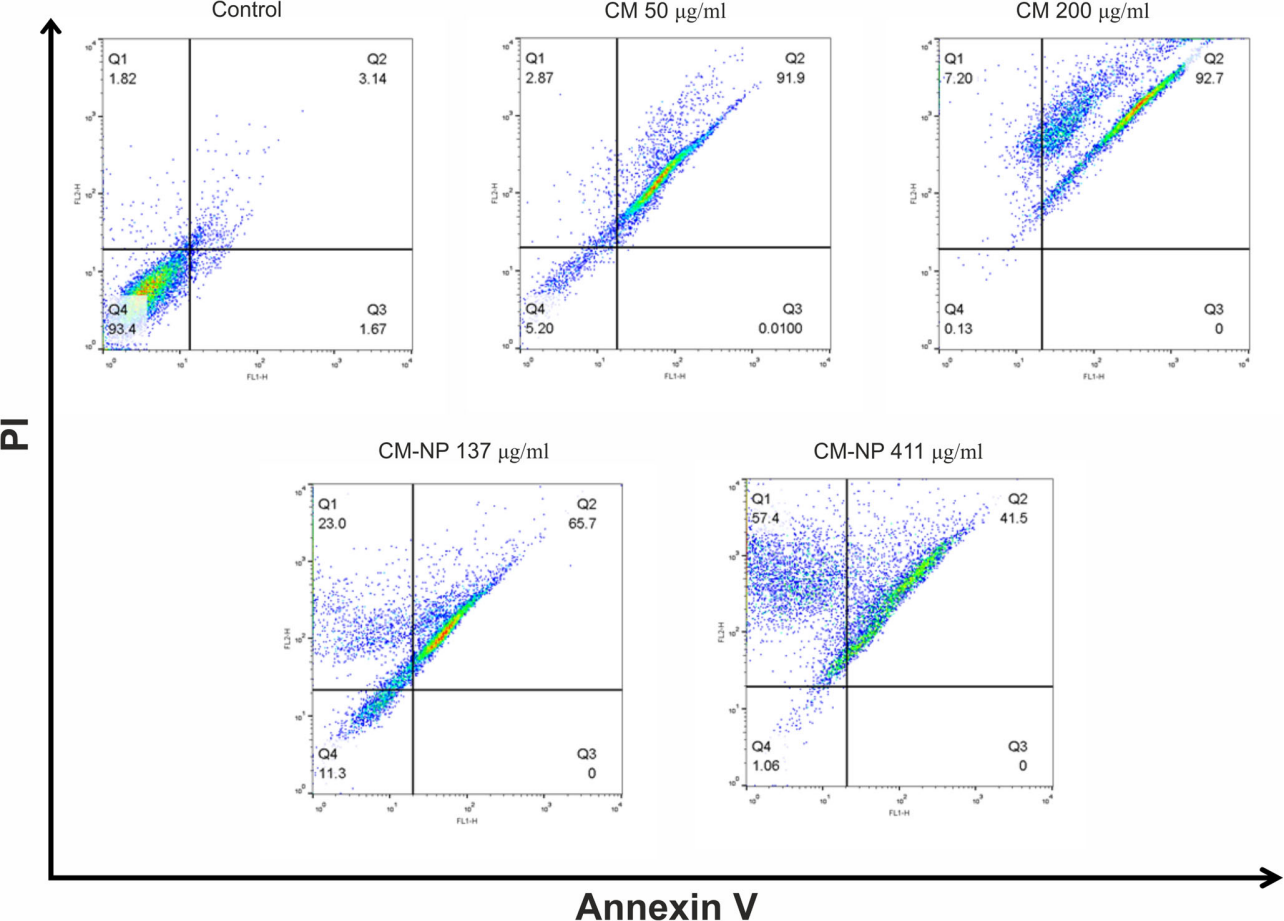
The effects of curcumin (CM) and curcumin-encapsulated noisome nanoparticle (CM-NP) on apoptosis of glioblastoma stem-like cells (GSCs). GSCs were double-stained with Annexin V/ propidium iodide and assessed by flow cytometry to determine apoptosis of GSCs treated with CM (50 and 200 μg/ml) and CM-NP (137 and 411 μg/ml). Untreated cells were indicated as control. Diagrams quarter 1 (Q1) to Q4 indicate necrotic, late apoptotic, early apoptotic, and live cells, respectively. Cells treated with CM-NP exhibited a higher amount of necrotic GSCs

To quantify GSCs death arising from necrosis and apoptosis, the cells treated with various values of curcumin and CM-NP were evaluated using Annexin V-FITC binding analysis and PI staining. As shown in Fig. 8, the number of apoptotic cells was 3% in the control group. Treatment with curcumin and CM-NP markedly increased the number of Annexin V-FITC-/PI-positive cells. The percentage of GSCs in the late apoptotic stage after application of curcumin at concentrations of 50 and 200 μg/ml was about 91.9 and 92.7%, respectively. In contrast to curcumin, the number of necrotic cells was significantly increased when GSCs were exposed to CM-NP. Flow cytometry analysis revealed that the percentage of late apoptotic and necrotic GSCs following administration of CM-NP at 137 μg/ml was 65.7% and 23%, respectively. CM-NP at 411 μg/ml enhanced the percentage of necrotic cells to 57.4% (Fig. 8). Our results indicate that CM-NP more efficiently induces cell arrest and increases cell apoptosis and necrosis in GSCs compared with free curcumin.

#### CM-NP Modulates Molecular Signaling of Apoptosis

**Fig. 9:**
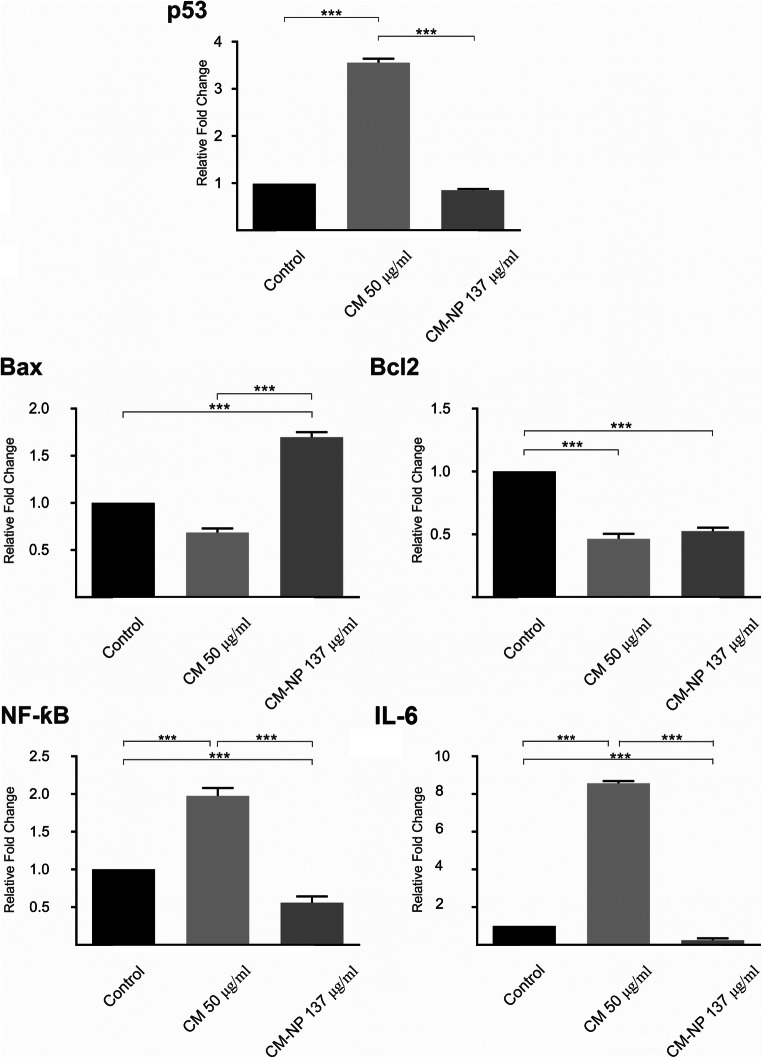
The effects of curcumin (CM) and curcumin-encapsulated noisome nanoparticle (CM-NP) on the mRNA expression levels of apoptotic markers p53, Bax, and Bcl2 as well as on chemokines NF-κB, and IL-6 of glioblastoma stem-like cells (GSCs). CM-NP significantly enhanced Bax and decreased Bcl2, NF-κB, and IL-6 compared with the control group. CM significantly increased the expression of p53 compared with the CM-NP and control groups, whereas CM-NP did not regulate p53. All data represent as the mean ± SD. Triple asterisks indicate *P* < 0.001

Alterations in the expression of p53, Bax, Bcl2, NF-κB, and IL-6 in GSCs were analyzed using real-time PCR. P53-mediated apoptosis and cell cycle arrest in cancer cells is suggested a desirable outcome of tumor therapy [40]. Curcumin significantly increased the expression level of P53, whereas no changes in the expression levels of p53 were observed after CM-NP treatment (Fig. 9; *P* < 0.001). Following 24-h treatment with CM-NP, the mRNA levels of Bax significantly increased compared with the free curcumin and untreated control group (Fig. 9; *P* < 0.001). Both curcumin and CM-NP significantly decreased the mRNA levels of Bcl2 compared with the control group (Fig. 9; *P* < 0.001). No significant differences were observed in the expression levels of Bcl2 between the curcumin and CM-NP groups. The NF-κB–IL-6 signaling pathway plays a crucial role in tumor cell proliferation and apoptosis [41]. The values of NF-ƙB and IL-6 following treatment with CM-NP were significantly lower than the curcumin and control groups (Fig. 9; *P* < 0.001). NF-ƙB and IL-6 levels were significantly higher in the curcumin-treated GSCs compared with the control group (Fig. 9; *P* < 0.001).

#### CM-NP Increases the Production of ROS

**Fig. 10:**
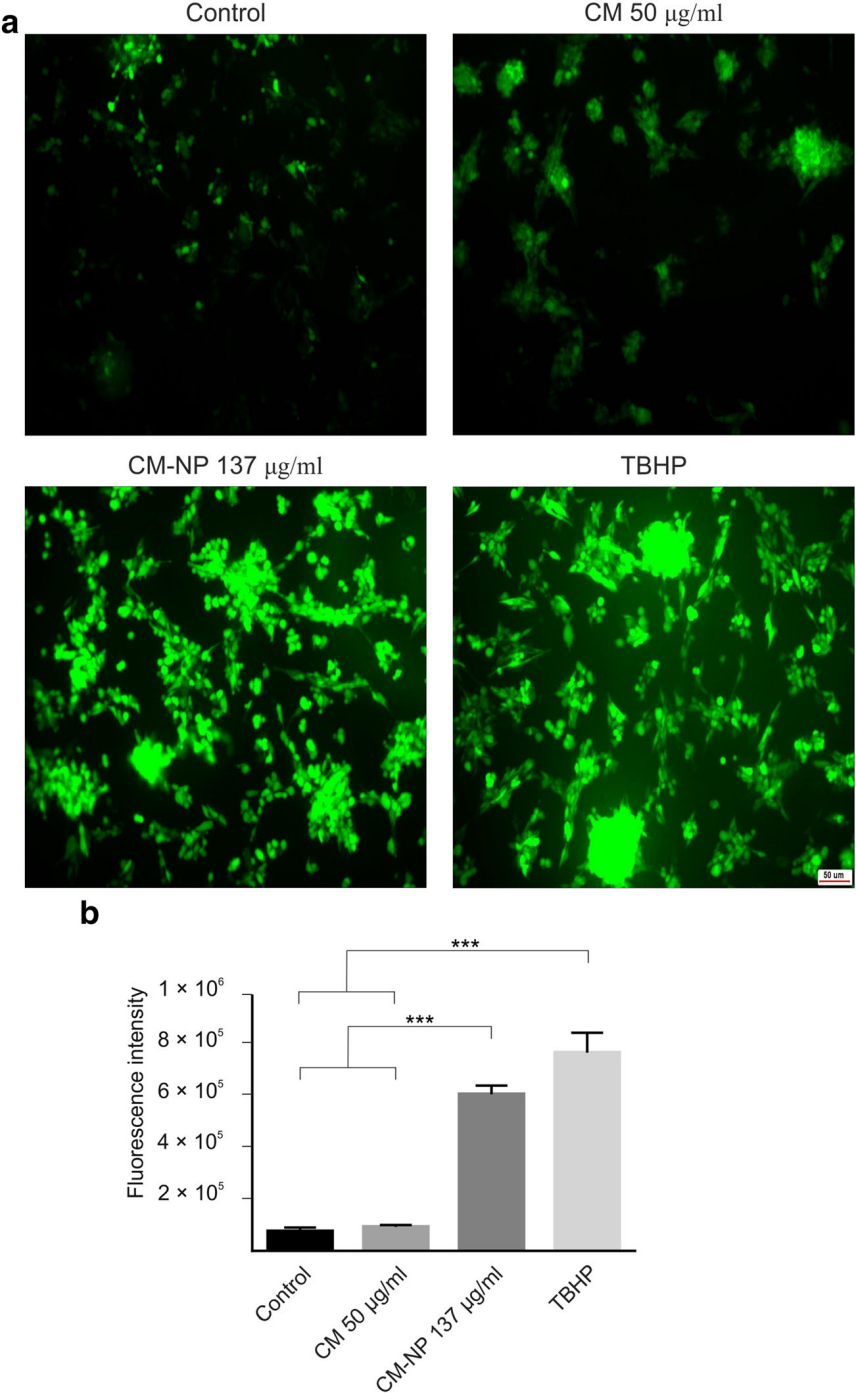
The effects of curcumin (CM) and curcumin-encapsulated noisome nanoparticle (CM-NP) on the generation of reactive oxygen species (ROS) in glioblastoma stem-like cells (GSCs). **a** Photomicrographs of curcumin-, CM-NP-, and tert-Butyl hydroperoxide (TBHP)–mediated ROS production in GSCs were obtained under fluorescence microscopy. **b** ROS induction in GSCs after 8 h application of CM, CM-NP, and TBHP as well as in the control group was determined by measuring fluorescent intensities in a microplate reader. Note a significant higher generation of ROS by application of CM-NP compared with the CM and control groups. Data are expressed as the mean ± SD. Triple asterisks indicate *P* < 0.001

Curcumin has been suggested to inhibit tumor growth via upregulation of ROS values [42]. The levels of ROS were significantly increased in the CM-NP-treated group compared with the curcumin and control groups (Fig. 10a and b; *P* < 0.001). Increasing the time of incubation and dose of CM-NP led to an increase in the levels of ROS in GSCs (data not shown). The effects of CM-NP on the levels of ROS were time-dependent.

### Migration and Invasion Properties

#### CM-NP Inhibits the Migration of GSCs

**Fig. 11:**
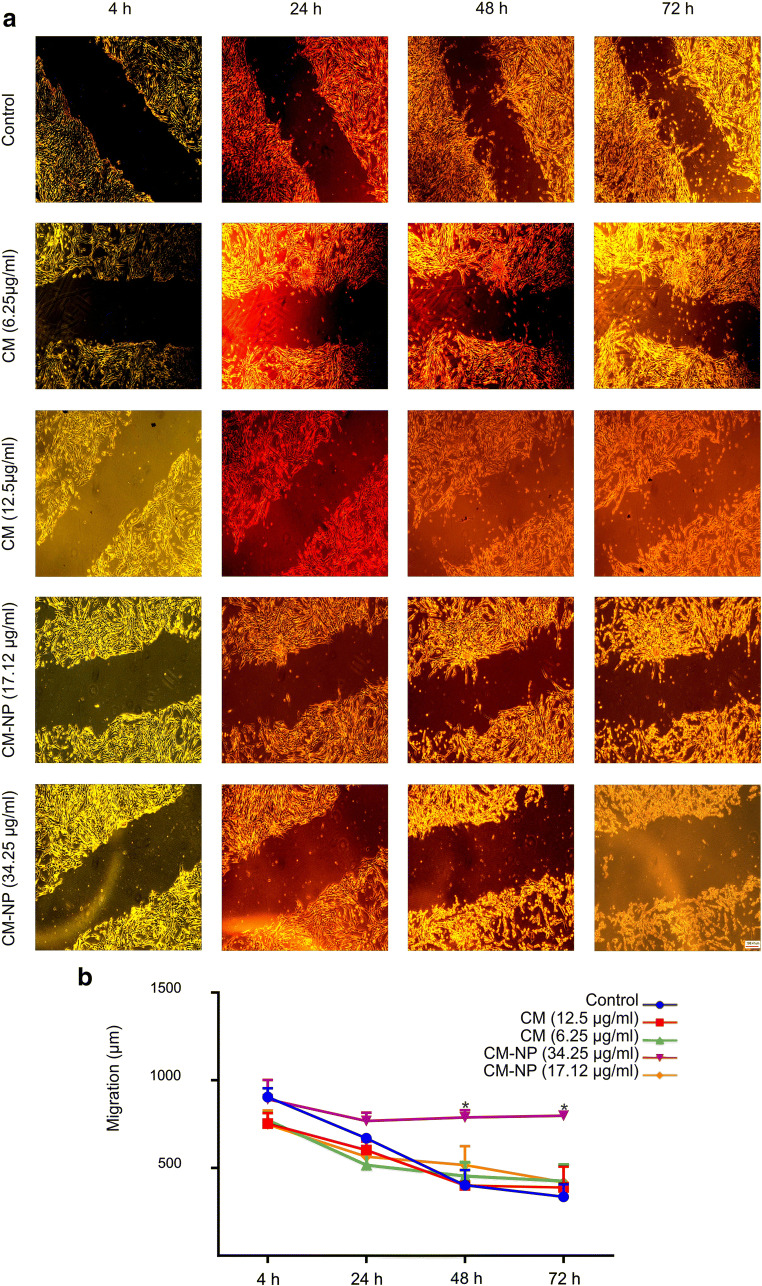
The effects of curcumin (CM) and curcumin-encapsulated noisome nanoparticle (CM-NP) on the migration of glioblastoma stem-like cells (GSCs). **a** Migration of GSCs was assessed by the wound healing method. After GSCs have reached 90% confluence, a scratch wound across a confluent monolayer of cultured cells was created on the cell surface using a micropipette tip and then CM (6.25 and 12.5 μg/ml) and CM-NP (17.12 and 34.25 μg/ml) were added. The cultures were incubated at 37 °C and images were captured with a microscope at 4, 24, 48, and 72 h of treatments. **b** The cell migration rate was estimated by the measurement of cell numbers within the wound region. CM-NP at 34.25 μg/ml significantly inhibited the migration of GSCs after 48 h and 72 h of application compared with the CM and control groups. The data are presented as mean ± SD. The asterisk indicates *P* < 0.05

To compare the inhibitory effects of free curcumin and CM-NP on GSC migration, we performed the scratch wound healing assay. CM-NP at 34.25 μg/ml significantly inhibited the migration of GSCs after 48 h and 72 h of application compared with the curcumin and control groups (Fig. 11a and b; *P* < 0.05). However, curcumin at different doses did not affect GSC migration. Therefore, our results showed that the inhibitory effects of curcumin increased when formulated in niosome nanoparticles.

#### CM-NP Suppresses MMP-2

**Fig. 12:**
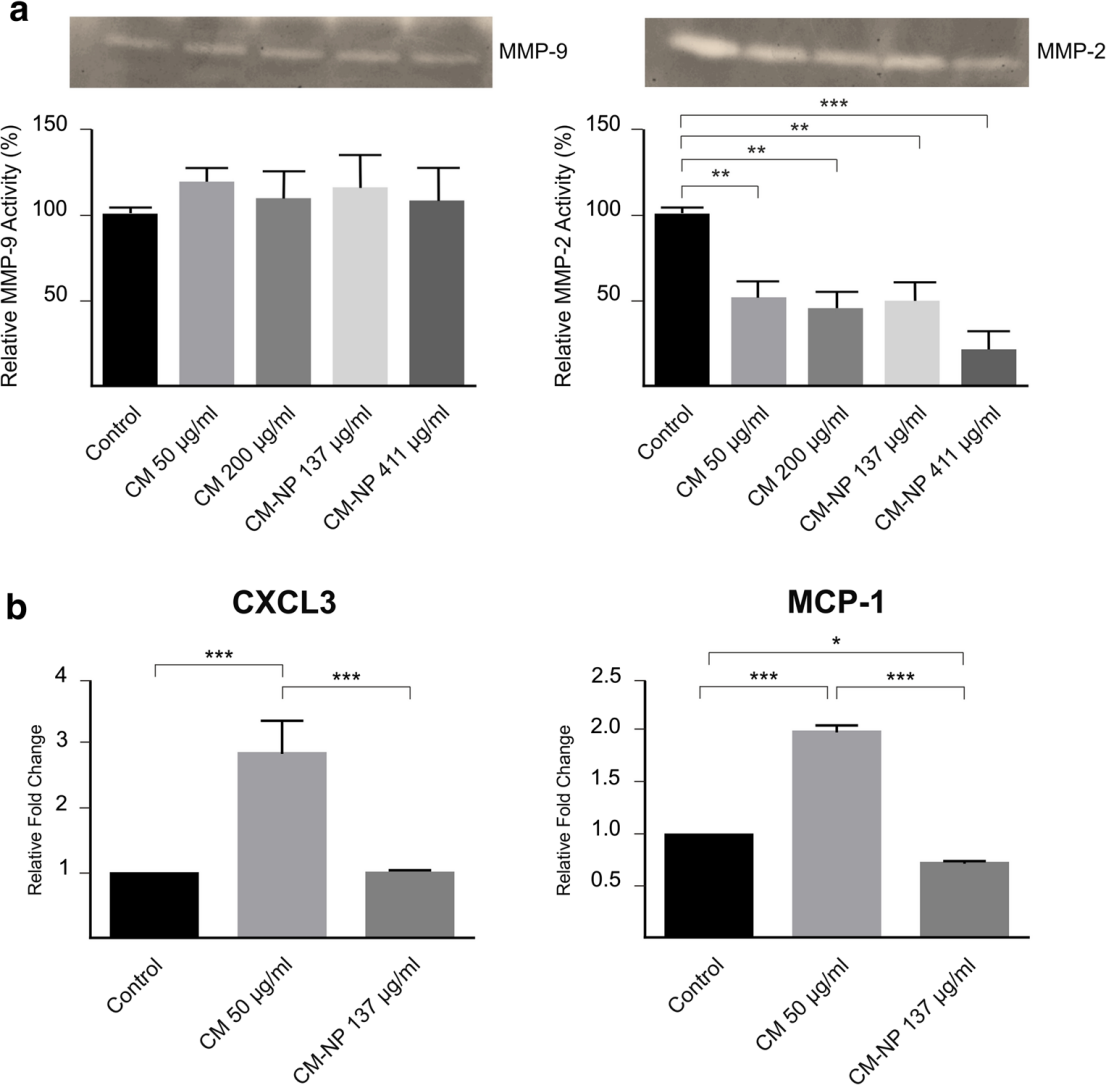
The effects of curcumin (CM) and curcumin-encapsulated noisome nanoparticle (CM-NP) on invasiveness of glioblastoma stem-like cells (GSCs). **a** Gelatinase zymogram and densitometry analysis of MMP-9 (left) and MMP-2 (right) secretion in conditioned media were detected by electrophoresis of soluble protein on a gelatin containing 10% polyacrylamide gel. Areas and relative intensities of gelatin-digested bands by MMP-9 and MMP-2 were quantified by densitometry and expressed as relative MMP-9 and MMP-2 activity compared with that of untreated cells. **b** The mRNA expression of CXCL3 and MCP-1 in GSCs treated with CM and CM-NP. Histogram represents mean ± SD. Triple asterisks indicate *P* < 0.001

MMP-2 regulates cancer cell behaviors, including tumor growth and migration [43]. To evaluate the effect of curcumin and CM-NP on the secretion of MMP-2 and MMP-9, GSCs were treated with different values of curcumin (50 and 200 μg/ml) and CM-NP (137 and 411 μg/ml). As indicated in Fig. 12a, treatment of GSCs with curcumin and CM-NP did not affect the secretion of MMP-9. In contrast, the secretion of MMP-2 was significantly reduced by application of curcumin at 200 μg/ml as well as by CM-NP at 411 μg/ml (*P* < 0.001).

#### CM-NP Inhibits GSCs Invasiveness

The effects of curcumin and CM-NP on the expression of CXCL3, a tumor metastatic marker, in GSCs were studied. As shown in Fig. 12b, application of CM-NP did not change the expression level of CXCL3 compared with the control group (Fig. 12b). However, curcumin significantly increased the mRNA expression level of CXCL3 compared with the control group (*P* < 0.001). Furthermore, the effects of curcumin and CM-NP on the expression of MCP1, a key regulator of cell migration and invasion in different cancer types, in GSCs were analyzed. Curcumin significantly increased the expression of MCP1 compared with the control group (*P* < 0.001). However, the expression of MCP1 significantly decreased after CM-NP treatment compared with the curcumin-treated cells (*P* < 0.001) and the control group (*P* < 0.05).

### CM-NP Changes the Characteristics of GSCs

#### CM-NP Disrupts Colony Forming

**Fig. 13:**
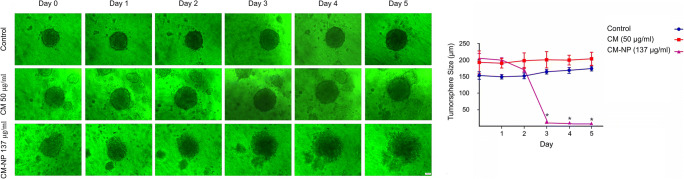
The effects of curcumin (CM) and curcumin-encapsulated noisome nanoparticle (CM-NP) on colony formation of glioblastoma stem-like cells (GSCs). The colony formation of GSCs was assessed in soft agar treated with CM (50 μg/ml) and CM-NP (137 μg/ml) for 5 days. CM-NP significantly decreased the size of tumor sphere and inhibited colony formation of GSCs. The data are presented as mean ± SD. The asterisk indicates *P* < 0.05

To evaluate the self-renewal ability of GSCs, the effects of curcumin (50 μg/ml) and CM-NP (137 μg/ml) on the sphere formation of GSCs seeded in soft agar were assessed for 5 days. A greater damage to the clonal sphere formation was observed after the administration of CM-NP on GSCs compared with the application of curcumin. In addition, a significant decrease in the diameter of tumorspheres was observed 3 days after the application of CM-NP (Fig. 13; *P < 0.05*).

#### CM-NP Decreases Cancer Stem Cell Markers

**Fig. 14:**
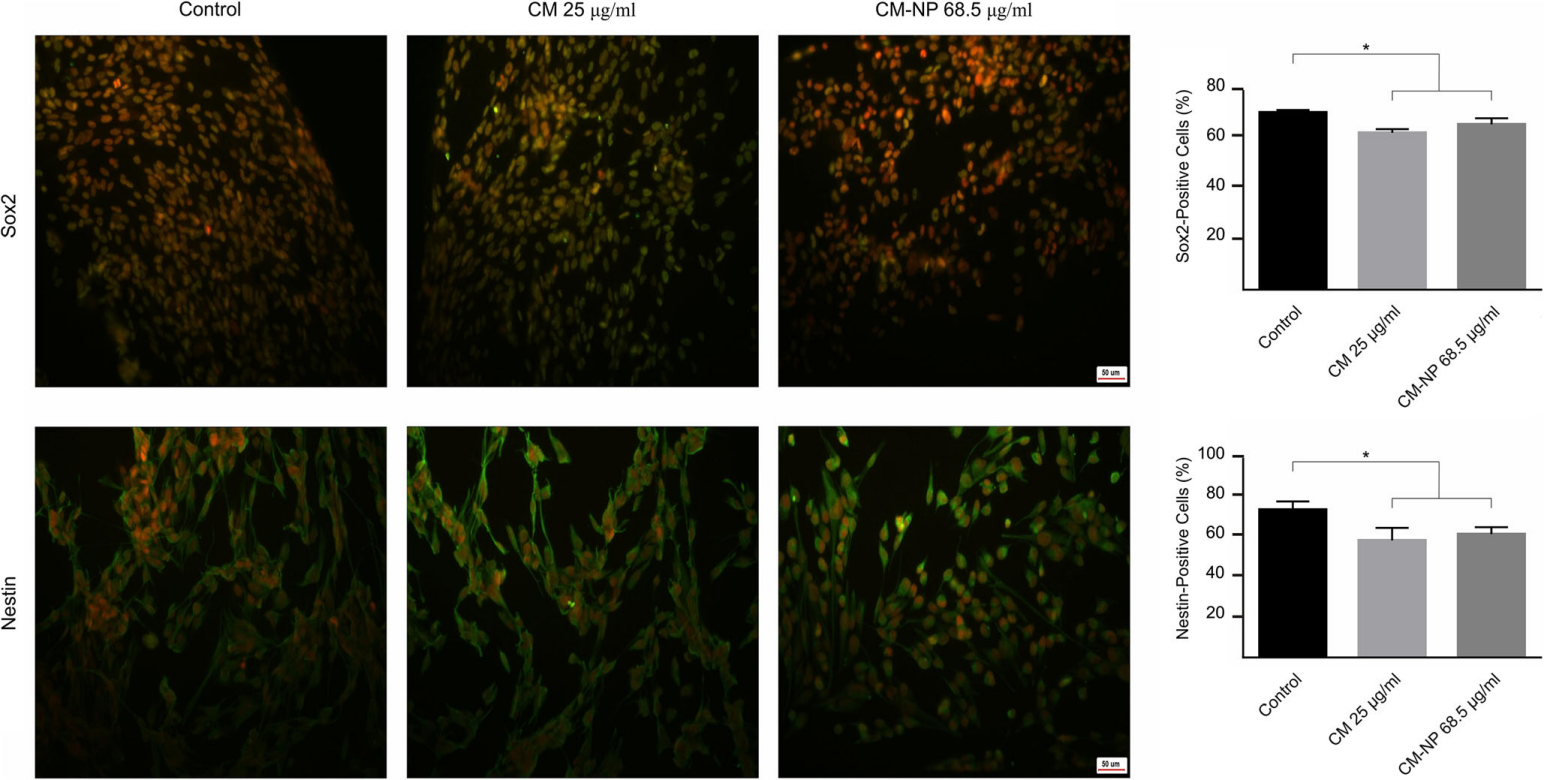
The effects of curcumin (CM) and curcumin-encapsulated noisome nanoparticle (CM-NP) on the expression of Sox2 and nestin in glioblastoma stem-like cells (GSCs). Quantitative analysis of the expression of Sox2 and nestin in GSCs revealed a significant reduction of these two markers after treatment with CM and CM-NP. Cells were stained for markers are shown in green and nuclei were stained with propidium iodide are shown in red. The data are expressed as mean ± SD. The asterisk indicates *P* < 0.05

The expression values of Sox2 and nestin, the GSCs markers [44], were investigated after the treatment of GSCs with curcumin and CM-NP. Our results demonstrated that the expression of Sox2 and nestin significantly decreased in GSCs following application of curcumin (25 μg/ml) and CM-NP (68.5 μg/ml) compared with the control group (Fig. 14; *P* < 0.05). There were no significant differences observed between CM-NP and curcumin effects on the expression of Sox2 and nestin (Fig. 14).

## Discussion

This in vitro study indicates that the cytotoxic effects of curcumin were markedly enhanced when loaded in niosome nanoparticles. CM-NP, in comparison with curcumin, more effectively reduced the viability, proliferation, and migration of GSCs isolated from human GBM through the induction of cell arrest and apoptosis as well as the enhancement of the expression of Bax, a pro-apoptotic marker. Furthermore, CM-NP considerably inhibited the GSC invasiveness, possibly via the inhibition MCP1, and tumor growth through the upregulation of ROS compared with curcumin. There was no significant difference between the inhibitory effects of curcumin and CM-NP on the expression of nestin and Sox2, neuroepithelial and cancer stem cell markers, in GSCs. Our data indicate that CM-NP, as a potentially safe compound with little effect on normal cells, exerts greater anti-tumor effects compared with free curcumin.

Over the last two decades, nanoparticle-based drug delivery systems have been developed for the treatment of various diseases, including brain tumors [45]. Various types of nanoparticle, including polymer-based, lipid-based, niosomal, viral, inorganic, and drug-conjugated, have been designed to target and demolish cancer cells [46]. Although different nanoparticles of curcumin formulations have been developed to improve its solubility, bioavailability, safety, and efficacy, none of those reaches optimal values [47]. Niosomal drug delivery approach enhances drug potency and reduces drug toxicity through keeping the compound localized at the site of application, increasing the drug stability, and improving drug penetrating capability [48]. In the present study, CM-NP has an average diameter of ~ 60 nm with a zeta potential of ∼ − 35 mV and ∼ 80% drug entrapment efficiency, which indicates an efficient encapsulation of curcumin [49]. The physical and chemical properties of CM-NP represent a permeable, stable, and inexpensive component for targeted curcumin delivery to cancer cells [31]. CM-NP exhibits a greater cytotoxic effect on GSCs compared with free curcumin. In keeping with our results, the anti-tumor effect of other types of curcumin nanoparticles, such as nanoliposome, was significantly greater than that of the free curcumin [26, 27, 50]. Niosome is among the promising drug carriers that act as a pool to release compounds in a steady, controlled, and sustained manner [51]. Inability to deliver active drug substances through the BBB to a large portion of GBM cells prevents efficient passage of drug molecules and contributes to treatment failure [52]. Due to low bioavailability, curcumin is not predicted to cross the BBB efficiently [53]. Engineered niosome encapsulation of curcumin has the potential to enhance the delivery of curcumin into the brain tumor [32, 54]. Curcumin and CM-NP at the IC50 value after 24 h (were 50 and 137 μg/ml) were applied on GSCs to study their anti-tumor effects. However, in the migration assay (wound healing stretch) and immunocytochemistry studies, lower concentrations of curcumin and CM-NP were administered to reduce their cytotoxicity for a better evaluation of cell behaviors, particularly when the compounds were used for longer than 24 h. For the evaluation of cell cycle arrest and apoptosis, alongside with IC50 concentrations of the substances, higher values were also tested. Our data suggest CM-NP at the IC50 value (137 μg/ml) as the optimal concentration for future studies.

GSCs are suggested to be responsible for tumor occurrence and progression as well as chemo- and radiation-therapy resistance [2]. In line with our data, several studies have shown that different curcuminoids, such as free curcumin [55], hydrazinobenzoylcurcumin [56], and demethoxycurcumin [20], exert cytotoxic effects on GSCs. In addition, application of liposomal or phytosomal curcumin inhibits the growth of GSCs [57, 58]. The present data indicate a greater effect of CM-NP on GSCs viability and proliferation compared with free curcumin. The greater inhibitory effects of CM-NP on GSCs proliferation could be due to a strong cell cycle arrest together with an effective apoptotic effect. It has been reported that curcumin can suppress GSCs and GBM growth via the inhibition of cell proliferation and survival, as well as regulation of diverse anti-tumor signaling pathways, including activation of apoptotic pathways, disruption of molecular signaling, inhibition of cell cycle, inhibition of angiogenesis, increase in ROS, recruitment of natural killer cells, enhancement of autophagy, and induction of differentiation of glioma-initiating cells [59, 60].

Treatment of GSCs with both curcumin and CM-NP modulates signaling pathway of apoptosis via enhancement of Bax and reduction of Bcl2. In contrast to CM-NP, curcumin also regulates p53 pathway. The tumor suppressor protein p53 is crucial in GBM prevention via modulating of a broad range of cellular responses, such as apoptosis of injured cells, reduction of angiogenesis, enhancement of genomic stability, regulation of cell cycle, and modulation of cell metabolism and tumor micromilieu [61]. Disruption of P53 pathway, presented in more than 90% of patients with GBM, is accompanied with a worse prognosis and is a crucial target for treatment approaches [62]. Previous investigations indicate that curcumin can cause apoptosis in both p53-dependent and p53-independent manners [63, 64]. Curcumin can promote the induction of apoptosis independently of p53 via phosphorylation of ROS, downregulation of anti-apoptotic protein Bcl-2, and enhancement of superoxide anion production [65]. The Bcl-2 expression has been reported in more than half of patients with GBM and Bcl-2 overexpression significantly enhanced tumor resistance against cytotoxic agents [66]. In addition, Bax activation inhibits proliferation and induces apoptosis of human GSCs [67]. In agreement with our findings, free curcumin increased Bax-Bcl-2 ratio and suppressed human GBM cell viability [68]. On the other hand, elevated NF-휅B activity and cytokine IL-6 release is correlated with cell viability in different GBM cell lines and with poor prognosis in patients with GBM [18]. NF-휅B plays a key role in both apoptotic and necrotic programmed cell death [69]. Inhibition of NF-휅B induces mitochondrial dysfunction with cell cycle arrest in the G2/M phase [18]. The present data support previous studies [68, 70] which indicate that the greater cytotoxic effect of CM-NP on GSCs compared with curcumin may exert through inhibition of the activation of NF-κB activity and the release of IL-6.

Reduced value of ROS is involved in the multistep oncogenesis process of GBM via the promoting of cell proliferation and survival as well as the activation of NF-κB. Enhanced ROS generation exerts anti-cancer effects via the induction of cell cycle arrest and apoptosis [71]. CM-NP exerts a significant higher effect on the production of ROD compared with curcumin. In agreement with a previous study, our data suggested that CM-NP may target GSC viability through the enhancement of ROS [55]. MMP-2 and MMP-9 play a crucial role in glioma invasiveness [72]. CM-NP suppressed the MMP-2 enzymatic activity, whereas it did not affect the MMP-9 activity in GSCs. Contrary to these findings, previous studies have suggested that curcumin is able to suppress the secretion of MMP-9 in GBM cell lines [15, 16]. In addition to MMP, chemokines play multiple roles in GBM invasion, proliferation, migration, and angiogenesis [73]. CXCL3 could bind to the chemokine receptor CXCR2 and regulates the progression and metastasis of malignant tumor [74, 75]. Evidence suggests that CXCL3 promotes the invasiveness of cancerous cells and may be a potential target for cancer therapy [74]. However, little is known about its function in glioblastoma [76]. Our study revealed that CM-NP could not inhibit CXCL3 in GSCs compared with the control group. However, CM-NP inhibited significantly the expression of MCP1 in GSCs compared with free curcumin. Interestingly, CCL2/MCP-1 (as the top-listed NF-κB-regulated gene) stimulated migration and invasion of GBM cells in a paracrine mode [76].

GSCs express both embryonic and neural progenitor cancer stem cell markers, including SOX2, nestin, and CD133. However, it should be noted that the expression of these markers is not restricted to one particular cell type in GBM [77]. A previous investigation has shown that a polymeric nanoparticle formulation of curcumin significantly inhibited clonogenic growth and decreased the number of CD133-positive stem-like population of GBM cells [28]. Here, both CM-NP and free curcumin significantly decreased the percentage of Sox2- and nestin-positive cells in GSCs. This may suggest the importance of these markers to identify GSCs and to explore the novel therapeutic approaches of GBM.

## Conclusion

The present study is the first to investigate the effect of a novel nano-niosome-loaded curcumin on GSCs. CM-NP was appropriately designed for the delivery of curcumin to GBM stem cells and the anti-tumor properties of curcumin significantly promoted when loaded in niosome nanoparticles. Our findings revealed that CM-NP more efficiently targets the viability, proliferation, and migration of GSCs than curcumin through multiple molecular mechanisms, including induction of cell cycle arrest, apoptosis, and ROS generation. CM-NP also alleviates GSC invasiveness via regulation of MCP-1. Our data suggested that nano-niosome could be used as an ideal carrier to deliver curcumin for possible therapeutic approaches of GBM. However, these findings should be interpreted with caution. Further in vivo investigations are warranted to confirm the anti-tumor effect of CM-NP on GBM and to validate their probable use in clinical trials.
